# How Challenge Demands Have Offsetting Effects on Job Performance: Through the Positive and Negative Emotions

**DOI:** 10.3389/fpsyg.2021.745413

**Published:** 2021-12-14

**Authors:** Qiong Wang, Aijing Xia, Wei Zhang, Zijun Cai, Xiyang Zhang, Xiaofei Teng, Jing Zhang, Jing Qian

**Affiliations:** ^1^School of Labor and Human Resources, Renmin University of China, Beijing, China; ^2^Business School, Beijing Normal University, Beijing, China; ^3^School of Psychology, University of Akron, Akron, OH, United States; ^4^Financial Department, Beijing University of Chemical Technology, Beijing, China

**Keywords:** challenge demands, positive emotions, negative emotions, job performance, offsetting effects

## Abstract

By combining the broaden-and-build theory of positive emotions (Fredrickson, 2001) and the transactional theory of stress (Lazarus and Folkman, 1984), this study examines how challenge demands (i.e., task complexity and time pressure) have dual effects on employees’ job performance through the mediating effects of positive and negative emotions. We collected data from 414 employees from three firms located in China, including two hi-tech firms and one financial firm. The results indicated that challenge demands (i.e., task complexity and time pressure) have an overall positive effect on employees’ job performance (i.e., task performance and contextual performance) by offsetting positive indirect effects with negative indirect effects. The theoretical and practical implications are also discussed.

## Introduction

People encounter many stressors at work, such as red tape, time constraints, and workload. To better understand the influences of stressors, Cavanaugh et al. (2000) introduced the challenge-hindrance framework. Hindrance stressors are those stressors that are likely to constrain personal development (Podsakoff et al., 2007). In contrast, challenge stressors are the stressors that will enhance one’s capability and promote personal growth (Podsakoff et al., 2007). This theory argues that different kinds of stressors would yield different outcomes, with hindrance stressors posing more negative effects and challenge stressors posing more positive effects. Numerous studies have relied on this lens to examine the relationship between stressors and a variety of important outcomes. One particularly important outcome is job performance, including both task and contextual performance, because it is fundamental for organizational effectiveness and individual development. Interestingly, existing research has got contrasting findings related to challenge stressors.

In detail, while scholars generally found negative relationships between hindrance stressors and job performance, the findings of challenge stressors are mixed. For example, Bakker et al. (2004) found that workload, a typical challenge stressor, negatively influenced in-role job performance. Webster et al. (2010) found that challenge stressors had a positive effect on job performance. Using meta-analyses, scholars also got different findings. LePine et al. (2005) found a positive relationship, but more recently, Mazzola and Disselhorst (2019) found insignificant relationships. The mixed results are troublesome because “one of the primary justifications researchers have used for embracing the CHM (challenge-hindrance) dichotomy is the supposed positive correlation between CS (challenge demands) and performance” (Mazzola and Disselhorst, 2019). Therefore, it is important to further clarify the relationship between challenge stressors and job performance.

Scholars have spent much effort doing so. Some argued that different appraisals would yield different outcomes. For example, González-Morales and Neves (2015) argued that seeing challenge stressors as opportunities would increase affective commitment, which in turn increases job performance; however, seeing them as a threat would increase distress, which in turn decrease job performance. Similarly, Ma et al. (2021) found that to what extent do people see challenge stressors as challenge influence their performance. Some focused on the individual difference. For example, Lu et al. (2016) found that people with high self-efficacy benefited more from challenge stressors. Lin et al. (2015) showed that conscientiousness was an important moderator. Some looked deeper into mechanisms. For example, Zhang et al. (2013) argued that justice also transmits the effects of stressors, besides strain. Despite all these important findings, the role of emotions has largely been ignored (an exception: Rodell and Judge, 2009). Thus, the current study aims to contribute to this line of research by showing that emotions could also explain why challenge stressors might have a mixed effect on job performance.

Specifically, built on the transactional stress model (Lazarus and Folkman, 1984), we examine the effects of two challenge stressors, task complexity (i.e., the mental load required by the job; Lin, 2010) and time pressure (i.e., a state of too much to do with too little time; Parker and DeCotiis, 1983). On one hand, challenge stressors promote personal growth and future gain (Podsakoff et al., 2007). They should increase positive emotions, which should make people willing to do better jobs. On the other hand, challenge demands pose potential threats to achieving desirable work outcomes (Podsakoff et al., 2007). These obstacles would increase negative emotions and make people more likely to withhold their job efforts (Lazarus, 1991) and thus do worse jobs. In short, we argue that challenge stressors would be related to job performance through two paths that have contrasting effects, which should explain the insignificant relationship between challenge stressors and job performance.

The current study will make at least two contributions to the existing literature. First, our research provides a new explanation of the mixed findings of the relationship between challenge stressors and job performance. While scholars tend to assume positive effects of challenge demands (Podsakoff et al., 2007), a recent quantitative review paper showed this might not be true. By decomposing the total effects into two contrasting indirect effects, we provide an emotional explanation of this finding. Second, we examine the positive and negative emotional mechanisms simultaneously. Previous studies have revealed some mechanisms through which challenge demands influence performance, such as psychological strain (LePine et al., 2005; Lang et al., 2007), work engagement (Karatepe et al., 2014), and organizational justice (Zhang et al., 2013), but they seldom considered contrasting explanations and compared them at the same time. As an exception, Rodell and Judge (2009) examined the unique effects of discrete emotions. We go beyond their studies by showing how more general emotional valences channel the effect of challenge stressors on individual performance. In sum, our research reminds scholars of the paradoxical nature of challenge stressors and the importance of seeing both sides of the coin through an emotional lens.

## Theoretical Background and Hypotheses

### Main Effects of Challenge Demand on Job Performance

Cavanaugh et al. (2000) proposed a two-dimensional framework to divide stressors into challenges and hindrances. It explained the rather inconclusive relationships between job stressors and employee performance by suggesting that hindrance stressors have detrimental effects on job performance whereas challenge stressors have the opposite effects.

Time pressure and task complexity were labeled as challenge stressors because these stressful demands are viewed as within employees’ locus of control. They are usually seen as factors that will promote personal growth and goal accomplishments (Cavanaugh et al., 2000; Wallace et al., 2009). By investing effort and time in the challenges, people have the potential to develop their abilities, achieve personal growth, and advance their careers (Lazarus and Folkman, 1984; LePine et al., 2005). Moreover, a myriad of empirical findings supports the idea that challenge stressors are positively related to performance (Wallace et al., 2009; Hon and Chan, 2013; Liu et al., 2013; Zhang et al., 2013; Lin et al., 2015; LePine et al., 2016; Lu et al., 2016; Karatepe et al., 2018; Van Laethem et al., 2019), which has also been supported by a meta-analysis (LePine et al., 2005). As a starting point to test our theoretical model, we expect to replicate the well-established positive relationship between challenging job stressors and job performance (i.e., task performance and contextual performance) in our current study. Accordingly, we propose the following:

***Hypothesis 1:*** *Challenge demands, as manifested by task complexity and time pressure, are positively related to employees’ job performance (i.e., task performance and contextual performance).*

### Indirect Effects of Positive and Negative Emotions on the Challenge Demands-Performance Link

The transactional stress model suggested that people appraise stressful factors, especially challenge demands, as potential opportunities for goal achievement, personal growth, and career development. This initial appraisal process could evoke positive emotions (Cavanaugh et al., 2000) because people are inclined to believe that they will obtain these valuable outcomes (e.g., promising career developments and prospects) if they meet these challenge demands with increasing effort (LePine et al., 2005; Rodell and Judge, 2009). For example, people are inclined to believe that they are capable of overcoming the constraints posed by higher levels of task complexity and time pressure at work. They are likely to believe that, with effective management, they will achieve work accomplishments. This process will subsequently trigger positive emotions and motivate employees to strive for the goals. Accordingly, we propose the following:

***Hypothesis 2:*** *Challenge demands, as manifested by task complexity and time pressure, are positively related to employees’ positive emotions.*

Although challenge demands will not necessarily trigger negative emotions (e.g., anxiety), a level of high job demands is likely to increase the level of uncertainty, which will make people feel threatened and hesitate to approach valued outcomes (Rodell and Judge, 2009). Lazarus (1991) suggested that not only actual threats lead to negative outcomes, but even potential threats could also lead to negative emotions, such as anxiety (Rodell and Judge, 2009), depression, and burnout (Lazarus and Folkman, 1984; Zhang et al., 2013). For example, task complexity and time pressure, although they are seen as challenge stressors, could also evoke uncertainties. Specifically, these stressors are likely to make employees doubt whether they can achieve work goals in time. From this perspective, challenge stressors are likely to trigger employees’ negative emotions. Indeed, it was supported by empirical evidence that challenge stressors will increase negative emotions. For example, Rodell and Judge (2009) found that challenge stressors were positively related to negative emotions (i.e., anxiety); Zhang et al. (2013) also found that challenge stressors were positively associated with psychological strains (e.g., anxiety, fear, and depression). Accordingly, we have the following hypothesis:

***Hypothesis 3:*** *Challenge demands, as manifested by task complexity and time pressure, are positively related to employees’ negative emotions.*

According to the transactional stress model, the emotional reactions that are generated from the challenge demands could influence subsequent behaviors at work, which is called the emotion-focused coping process (Lazarus and Folkman, 1984). These coping behaviors will be able to relieve the emotional reactions to stressful experiences. It means that as a process of emotion-focused coping, challenge demands (i.e., task complexity and time pressure) are likely to evoke more efforts to cope with stress, which will ultimately lead to better task performance and contextual performance. However, not all coping strategies will result in beneficial outcomes, and there are circumstances in which stress coping could lead to deviant behaviors (Weiss and Cropanzano, 1996; Spector and Fox, 2002), and therefore have negative implications to task and contextual performance.

**FIGURE 1:**
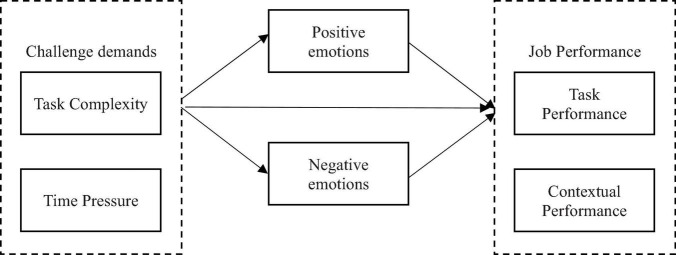
Theoretical model.

As shown in Figure 1, we expected that positive emotions would be positively associated with job outcomes that consisted of task and contextual performance. According to the emotion-focused coping process (Lazarus and Folkman, 1984), employees with positive emotions would be more willing to increase their efforts to fulfill task performances or engagement in helping behaviors. Indeed, numerous studies in previous research support the argument that people who experienced positive emotions were inclined to be more actively engaged in improving their task performance (e.g., Sonnentag and Starzyk, 2015) or contextual performance (e.g., Rodell and Judge, 2009). Specifically, Sonnentag and Starzyk (2015) argued that the positive emotional states triggered by challenge stressors would stimulate employees’ behaviors of implementation, which was viewed as a positive predictor of task performance. In addition, Rodell and Judge (2009) posit that employees who experienced positive emotions were more likely to help others at work and behave in more collaborative ways toward organizational goals and developments, which is labeled as contextual performance.

In contrast to positive emotions, we expected that negative emotions would be negatively associated with job performance (i.e., task performance and contextual performance). Negative emotions caused by challenge demands are likely to make people avoid confronting challenges (Lazarus, 1991). People have the innate motivation to avoid stimuli that make them anxious or scared. This is because avoidance or withdrawal can provide temporary relief for the stress and decrease experienced negative emotions (Roth and Cohen, 1986). For example, a previous study suggested that negative affective thoughts stimulated by perceived stress were negatively associated with employees’ performances (Edwards et al., 2014). Moreover, Rodell and Judge (2009) found that negative emotions (i.e., anxiety) triggered by challenge stressors were negatively related to employees’ citizenship behaviors. Accordingly, we propose the following:

***Hypothesis 4:*** *Positive emotions are positively associated with employees’ job performance (i.e., task performance and contextual performance).*

***Hypothesis 5:*** *Negative emotions are negatively associated with employees’ job performance (i.e., task performance and contextual performance).*

***Hypothesis 6:*** *Challenge demands, as manifested by task complexity and time pressure, will have a positive indirect relationship with job performance (i.e., task performance and contextual performance), as mediated by positive emotions.*

***Hypothesis 7:*** *Challenge demands, as manifested by task complexity and time pressure, will have a negative indirect relationship with job performance (i.e., task performance and contextual performance), as mediated by negative emotions.*

### The Overall Effect

Fredrickson (1998, 2001) posited the offsetting hypothesis in the broaden-and-build theory of positive emotions, suggesting that positive emotions can undo the lingering effects of negative emotions, that is, positive emotions can restrain or offset subsequent negative effects or outcomes from negative emotions. Given the narrow individual instant mind-action range of negative emotions and the ability of positive emotions to broaden it. Fredrickson (1998) argued that positive emotions may play a role in undoing negative lingering effects, similar to an antidote. Indeed, Fredrickson and her colleagues observed and tested this undoing effect both in a laboratory study and in-field research (Fredrickson et al., 2000, 2003). For example, Fredrickson et al. (2000) conducted a laboratory experiment and found that, compared with those who watched neutral or sad films, participants who saw contentment-eliciting and amusing films experienced faster cardiovascular recovery. Subsequently, Fredrickson et al. (2003) examined 46 U.S. college students who experienced the 9–11 terrorist attacks, and their findings indicated that positive emotions in the aftermath of crises helped alleviate negative emotions (e.g., depression) in resilient people. From this perspective, although numerous prior studies emphasized the positive effects of challenge demands on performance, negative emotions triggered by challenge demands cannot be ignored because they consistently exist in a stressful context. Despite the existence of the positive effect of challenge stressors on performance, the negative emotions arising from the stressors cannot be ignored. However, positive emotions will have an offsetting effect on negative emotions (Fredrickson, 1998, 2001; Fredrickson et al., 2003). Accordingly, we propose the following:

***Hypothesis 8:*** *Challenge demands, as manifested by task complexity and time pressure, will have a positive overall relationship with job performance (i.e., task performance and contextual performance), as mediated by a positive indirect effect of positive emotions and a negative indirect effect of negative emotions.*

## Materials and Methods

### Participants and Procedure

Data were collected from three firms located in China, including two high-tech firms and one financial firm. We chose these two firms because task complexity and time pressure are common in these firms. For example, employees in high-tech firms need to solve complex technology problems. Those in the financial firm usually face time dues to finish reports. Thus, these firms provide suitable research context for the topic.

In China, formal ethical approval is not necessary for data collection, but strictly following ethical principles is required and a “must-do.” In detail, our survey does not contain any danger to participants. We did not collect any secretary information. Participants were free to join and quit. Data were carefully stored in the computer of one research team member. No one, except our research them, has access to the data. We did not reveal any private information. The data were only for this study and thus only general trends and relationships were presented.

We approached the head of human resource departments and told them our research topic. After getting their consent, we distributed invitations to employees through emails via the help of HRs in these firms. In the email, we told employees that we were running a research project about stressors at work and were interested in their impacts. Employees are free to join our project and what they need to do is just filling surveys. 486 employees agreed to participate. We then sent these employees links to our questionnaire and told them that they could quit at any time without any expenses. Finally, 414 valid questionnaires were completed and returned. Compared with previous studies about challenge stressors (e.g., Zhang et al., 2013), the sample size is satisfying.

Among the participants, 40.8% were men, 47.3% were married, 62.8% held a bachelor’s degree, and 22.2% held a college degree. The average age was 30.07 years (*SD* = 8.76). The average work tenure was 8.22 years (*SD* = 8.29), and the average current organizational tenure was 4.59 years (*SD* = 5.50). Besides, as shown in Table 1, we observed enough variances in all interesting variables. Thus, the sample is representative at a satisfying level.

**TABLE 1:** Means, standard deviations, reliabilities, and correlation of constructs*.

No	Construct	Mean	Std. dev.	Composite reliability	Correlation of constructs and average variance extracted							
					1	2	3	4	5	6	7	8
1	Challenge demands	4.920	1.015	0.844	**0.855**							
2	Contextual performance	5.117	1.036	0.813	0.380	**0.722**						
3	Job performance	5.422	0.827	0.872	0.319	0.895	0.879					
4	Negative emotions	4.114	1.440	0.914	0.316	0.007	–0.059	**0.799**				
5	Positive emotions	5.324	1.047	0.897	0.215	0.505	0.547	–0.040	**0.770**			
6	Task complexity	5.263	1.106	0.827	0.878	0.407	0.388	0.202	0.323	**0.784**		
7	Task performance	5.726	0.841	0.869	0.191	0.578	0.877	–0.123	0.465	0.291	**0.790**	
8	Time pressure	4.577	1.265	0.833	0.834	0.235	0.144	0.348	0.041	0.476	0.019	**0.793**

*N = 414. Diagonal elements in the correlation of constructs matrix are the square root of the average variance extracted. For adequate discriminant validity, diagonal elements should be greater than corresponding off-diagonal element. AVE values are presented along the diagonal.

### Measures

The measures we used were originally constructed in English. We performed standard translation and back-translation procedures (Brislin, 1980) to ensure the equivalence of the survey instruments and to mitigate the effect of cultural differences. We evaluated challenge demands as a second-order formative construct because task complexity and time pressure contribute to the demands while examining other constructs as reflective. Additionally, to simplify our model, we tested job performance as a second-order construct of the first-order variables, which were task performance and contextual performance. Following the method of Miyamoto and Ryff (2011), we asked participants to complete the questionnaires based on their experiences at work during the past 30 days.

#### Task Complexity

We used a 3-item scale (Lin, 2010) to measure task complexity. Employees were asked to indicate the degree to which they agreed with the description of their work experience during the past 30 days (1 = strongly disagree to 7 = strongly agree). One sample item is “My job is a very complex one.”

#### Time Pressure

We adapted the 3-item scale (Lin, 2010) to measure time pressure. Employees were asked to rate the extent to which they agreed with the description of their work experience during the past 30 days (1 = strongly disagree to 7 = strongly agree). One sample item is “I have no sufficient time to think carefully about my job during the past 30 days.”

#### Challenge Demands

We used task complexity and time pressure as two measurements for challenge demands. Given that challenge demands were a second-order construct of task complexity and time pressure, we used the item grouping method to measure these factors (Landis et al., 2000) by grouping the items of task complexity into one group and the items of time pressure into another group.

#### Positive and Negative Emotions

Positive and negative emotions were measured using the positive and negative affect scale (PANAS) (Watson et al., 1988), which was largely applied to measure positive and negative emotions (Aaker et al., 2008; Spencer-Rodgers et al., 2010; Moeller et al., 2018) because of its reliability and validity. We applied a short version of PANAS with 6 items of positive emotions and 6 items of negative emotions. Employees were asked to indicate the extent to which they agreed with the description of their work experience during the past 30 days (1 = never to 7 = always). A sample item of positive emotions is “Enthusiastic,” and a sample of negative emotions is “scared.”

#### Task Performance

We adapted the 4-item scale developed by Van Dyne and LePine (1998) to measure task performance. Employees were asked to rate the extent to which they agreed with the description of their task performance during the past 30 days (1 = strongly disagree to 7 = strongly agree). One sample item is “I fulfill the responsibilities specified in my job description.”

#### Contextual Performance

We used the 5-item scale developed by Borman and Motowidlo (1997) to measure contextual performance. Employees were asked to indicate the extent to which they agreed with the description of their contextual performance during the past 30 days (1 = strongly disagree to 7 = strongly agree). One sample item is “Helping and cooperating with others.”

#### Analytic Strategy

Our hypothesized model contained first and second-order constructs. To test our model, we used partial least squares, a structural equation modeling (SEM) method, which allows researchers to measure second-order variables and formative and reflective constructs mixed within a model and allows the integration of measurement and structural models (Bollen, 1989). We used the software SmartPLS3.2.8 (Ringle et al., 2015) to test our model. First, we examined the hypothesized links between the indicators and latent constructs with the measurement model. Second, we estimated the significance of the hypothesized paths between the study variables using bootstrapping with subsamples of 5,000 to ensure the stability of the results.

**FIGURE 2:**
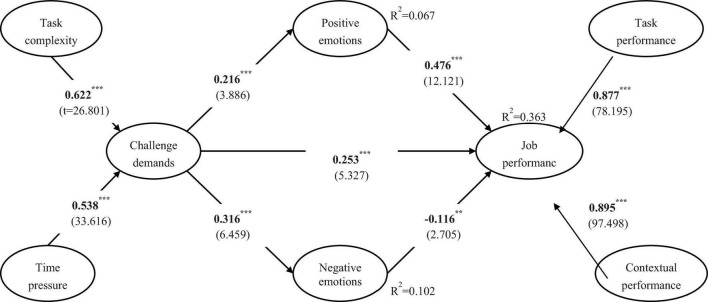
Results. Path coefficients with *t*-values in parentheses after controlling Marker variables including education, industry, and locations. **Significant at 0.01 level. ***Significant at 0.001 level.

To note, since we adopted the self-reported method to collect data, the results might be biased due to the common method variance. To examine whether the potential bias influenced our results, we employed the marker variable technique (Lindell and Whitney, 2001). In our research, we designed education, industry, and locations as the marker variables, since to the best of our knowledge, no theoretical arguments have been proposed to support the notion that these variables are related to the other variables in our model. Table 2 presents the results of path coefficients before and after controlling the marker variables, which showed that there were no significant distinctions between them. Figure 2 shows the results after adding the marker variable as an additional control variable in the estimated model, and these results confirmed that our findings remained unaltered. In summary, the results mentioned above show that the common method bias did not pose a serious threat.

**TABLE 2:** Path coefficients before and after controlling CMB.

Construct	Before							After						
	CP	JP	NE	PE	TC	TP1	TP2	CD	CP	JP	NE	PE	TC	TP2
CD		0.250	0.319	0.210	0.869		0.843			0.253	0.316	0.216		
CP														
JP	0.895					0.877			0.895					0.877
Marker										–0.087	0.046	–0.144		
NE		–0.119								–0.116				
PE		0.490								0.476				
TC								0.622						
TP2														
TP1								0.538						

*CP, contextual performance; JP, job performance; NE, negative emotions; PE, positive emotions; TC, task complexity; TP1, task performance; TP2, time press.*

## Results

### Measurement Model

To assess reliability and validity, we conducted a PLS to calculate a series of composite reliabilities of indicators, and average variance extracted (AVE) using the typical method (Barclay et al., 1995; Chin and Marcoulides, 1998). Table 1 presents the results of the reliabilities and correlation of constructs. As shown in the table, the composite reliabilities of the constructs, interpreted like Cronbach’s alpha internal consistency reliability estimates, are all above.70, which is considered acceptable for research (Fornell and Larcker, 1981). The AVE calculates the variance estimated by the indicators relative to measurement error (Fornell and Larcker, 1981), and the AVEs of the current study are all greater than.50 as recommended (Barclay et al., 1995).

To evaluate distinctiveness and convergence, we examined the correlation of variables (see Table 1) and factor loading (see Table 3). Fornell and Larcker (1981) suggested that when the square root of each variable’s AVE is greater than the correlation of the variable to other latent variables, the correlation of variables provides support for discriminant validity. Additionally, an indicator’s factor loading also allows us to evaluate discriminant validity (Chin and Marcoulides, 1998) by loading higher on the construct of interest than on any other variable. Because of a low factor loading for one item, contextual performance was reduced to a four-item scale.

**TABLE 3:** Results of total, indirect (specific and total), and direct effect.

Relationships	Original sample (O)	Mean	Standard deviation (STDEV)	T statistics (|O/STDEV|)	*P* values
**Total effects**					
CD - > JP	0.320	0.320	0.052	6.199	0.000
TC - > JP	0.199	0.200	0.035	5.753	0.000
TP1 - > JP	0.172	0.171	0.026	6.621	0.000
TC - > TP2	0.174	0.175	0.031	5.700	0.000
TP1 - > TP2	0.151	0.150	0.023	6.561	0.000
TC - > CP	0.178	0.179	0.031	5.679	0.000
TP1 - > CP	0.154	0.154	0.024	6.528	0.000
**Specific indirect effects**					
CD - > PE - > JP	0.103	0.104	0.027	3.839	0.000
CD - > PE - > JP - > TP2	0.090	0.091	0.024	3.806	0.000
TC - > CD - > PE - > JP - > TP2	0.056	0.057	0.016	3.597	0.000
TP1 - > CD - > PE - > JP - > TP2	0.048	0.049	0.012	4.030	0.000
CD - > PE - > JP - > CP	0.092	0.093	0.024	3.816	0.000
TC - > CD - > PE - > JP - > CP	0.057	0.058	0.016	3.606	0.000
TP1 - > CD - > PE - > JP - > CP	0.050	0.050	0.012	4.042	0.000
CD - > NE - > JP	–0.037	–0.037	0.015	2.464	0.014
CD - > NE - > JP - > TP2	–0.032	–0.032	0.013	2.460	0.014
TC - > CD - > NE - > JP - > TP2	–0.020	–0.020	0.008	2.508	0.012
TP1 - > CD - > NE - > JP - > TP2	–0.017	–0.017	0.007	2.396	0.017
CD - > NE - > JP- > CP	–0.033	–0.033	0.013	2.466	0.014
TC - > CD - > NE - > JP - > CP	–0.020	–0.021	0.008	2.514	0.012
TP1 - > CD - > NE - > JP - > CP	–0.018	–0.018	0.007	2.401	0.016
**Total indirect effects**					
CD - > JP	0.066	0.067	0.032	2.060	0.039
TC - > JP	0.199	0.200	0.035	5.753	0.000
TP1 - > JP	0.172	0.171	0.026	6.621	0.000
TC - > TP2	0.174	0.175	0.031	5.700	0.000
TP1 - > TP2	0.151	0.150	0.023	6.561	0.000
TC - > CP	0.178	0.179	0.031	5.679	0.000
TP1 - > CP	0.154	0.154	0.024	6.528	0.000
**Direct effect**					
CD - > JP	0.253	0.253	0.048	5.327	0.000

### Structural Model

Figure 2 presents a graphic depiction of the PLS results after controlling the marker variables. As shown in Figure 2, all paths are significant with the model accounting for 6.7% of the variable in positive emotions, 10.2% of the variable in negative emotions, and 36.3% of the variable in job performance. The results in Figure 2 support the proposition that challenge demands (i.e., task complexity and time pressure) can contribute to employees’ positive emotions and their negative emotions. As expected, employees’ positive emotions (positive) and negative emotions (negative) are each related to their job performance (i.e., task and contextual performance) while challenge demands have a positive relationship with employees’ job performance, which provides the first support that challenge demands have dual effects on individual behavioral outcomes. In summary, the results mentioned above support hypotheses 1 through 5.

### Indirect Effect Tests

As shown in Table 3, all the total effects are significant between the independent variables and dependent variables, and the specific indirect effects are also significant. Moreover, the direct effect of challenge demands on job performance is significant. Altogether, the above results provide support for the partially mediating effect of positive and negative emotions on the relationship between challenge demands and performance and further support for the dual effects of challenge demands on employees’ job performance. Therefore, the results mentioned above support hypotheses 6 through 8. In summary, all of the hypotheses are supported by the results.

## Discussion

The current study aims to answer why there are mixed findings of the relationship between challenge stressors and job performance. Drawing on the broaden-and-build theory of positive emotions (Fredrickson, 2001) and the transactional stress model (Lazarus and Folkman, 1984), we proposed one reason is that challenge stressors have contrasting effects on emotions, which in turn influence job performance. Using data collected from three firms, we showed that (1) challenge stressors, as manifested by time pressure and workload, had a positive indirect effect on job performance through positive emotions; (2) simultaneously, challenge stressors had a negative indirect effect on job performance through negative emotions; (3) due to that the strength of the positive indirect effect was stronger than that of negative one, the total effect was positive. The results carry important implications for both research and practices.

### Theoretical Implications

The current study mainly offers three theoretical contributions. First, we provide further evidence that challenge stressors are emotional triggers. To understand why challenge stressors influence job performance, scholars have relied on the different theoretical lens and revealed different mechanisms. Some are more focused on cognitive factors, especially how people appraise the stressors (e.g., Ma et al., 2021). Some more focused on motivational parts. For example, Webster et al. (2010) showed significant influence on self-efficacy. Drawing on the transactional stress model (Lazarus and Folkman, 1984), we showed that challenge stressors could also trigger emotions: Both positive and negative ones simultaneously. Previous studies have proposed that challenge stressors could elicit emotions, but they mainly examined one emotional state (e.g., Webster et al., 2010) and focused on specific ones (Rodell and Judge, 2009). As we showed, challenge stressors could lead to emotions in opposing valence. This makes challenge stressors different from hindrance stressors and probably unique from other variables that mainly contain emotional meaning in one valence (e.g., abusive supervision).

Second, we help answer why challenge stressors might not always increase job performance. In theory challenge stressors have positive implications for job performance (Mazzola and Disselhorst, 2019), existing studies did not always support this key proposition. To resolve the mismatch between theory and empirical findings, previous studies have mainly focused on cognitive (e.g., Ma et al., 2021) and motivational (e.g., Zhang et al., 2013) reasons. We extend this line of research by adopting an emotion-focused perspective. Integrating the effects on emotions and the broaden-and-build theory (Fredrickson, 2001), we showed the double-edged effects of challenge stressors: They simultaneously elicit both positive and negative emotions, which in turn impact job performance in contrasting ways. The results provide an emotion-based explanation about why challenge stressors might not always benefit job performance: The positive and negative emotions contain offsetting indirect effects.

Third, we further reveal the complex nature of challenge stressors. One intriguing characteristic of challenge stressors is that, unlike hindrance stressors, besides being demanding, they also provide opportunities for employees. Because of these seeming conflicting features, previous studies have tried to reveal the inherent complexity of challenge stressors. For example, using meta-analyses, LePine et al. (2005) showed that challenge stressors increase both strains and motivation. Similarly using meta-analyses, Podsakoff et al. (2007) showed that challenge stressors, besides the indirect effect through strain, also directly impact outcome variables. The current study extends this line of research by showing that challenge stressors could increase positive and negative emotions simultaneously. This finding further informs scholars that to better understand challenge stressors, it is better to take a holistic perspective to consider possible contrasting mechanisms, rather than merely focusing on effects in one direction. Otherwise, we might get inaccurate findings and biased understandings.

Besides, while not being our main focus, the current study also enriches the understandings of the relationship between positive and negative emotions. Our results could increase the confidence in the argument that positive and negative emotions could coexist rather than exclude each other. More importantly, we showed that positive emotions had a stronger influence than negative emotions when people face challenge stressors: The total effect of challenge stressors on job performance was positive in our study. The findings move forward the discussion about the relative importance of different emotions in organizations and show the importance to consider different emotions at the same time, rather than focusing on a specific emotion. Furthermore, the findings showed one way to manage the detrimental effects of negative emotions: By eliciting positive emotions at the same time.

### Practical Implications

The current study also provides the following practical implications. First, managers should be aware that challenge stressors might not always be beneficial. Challenge stressors, such as time pressure and high complex tasks, are not uncommon in current workplaces. One underlying reason is the myth that they could improve organizational effectiveness. However, as we showed, this might not always be true. Although the current study showed a positive net effect, it is possible that in other situations the negative sides would become stronger, such as for those who are low in emotional stability. Besides, negative emotions could introduce other problems. Thus, managers should take in mind that there are both pros and cons of challenge stressors and be more careful before introducing these stressors into work.

Second, managers should try to manage the negative emotions elicit by challenge stressors. Negative emotions offset the positive indirect effects of challenge stressors, prohibiting managers from capturing the full benefits. We argue that one reason that negative emotions arise is because of uncertainties. Thus, organizations could provide more fair practices to employees to help them manage the uncertainties (Lind and van den Bos, 2002). Other theories also provide some practical wisdom. For example, according to the job demand and resource model (Demerouti et al., 2001; Demerouti, 2007), providing more resources, such as autonomy and support, could help people manage the stressful side of challenge stressors. According to dual-system theory (Evans, 2008), more deliberative thinking could mitigate the detrimental effects of negative emotion. Managers could improve their work design to give employees more cognitive resources (Parker, 2014).

Third, managers should try to enhance the indirect effect through positive emotions. As we found, challenge stressors increased positive emotions, possibly because they provide opportunities to employees. Organizations could more emphasize the challenges and future gains of these stressors, positively encouraging employees to overcome challenges by self-learning. They could also train employees to be more skilled to surmount job challenges. Besides, organizations could also use the feedback system to enhance employees’ motivation to grasp the opportunities. According to feedback intervention theory (Kluger and DeNisi, 1996), timely performance feedback that focuses on task-related details could help people better learn from their jobs, so that challenge stressors could be seen as developmental opportunities.

In short, as our research shows, challenge stressors have contradicting effects on job performance through positive and negative emotions. Managers should be aware of this phenomenon and try to maximize benefits through exaggerating the positive effects through positive emotions and reducing costs through mitigating the negative effects through negative emotions.

### Study Limitations and Future Directions

Scholars could extend our findings in several ways. First, they could examine the boundaries of the dual mediation effects. As we mentioned in the above section, personalities, job designs, and human resource management systems might influence the relative strength of these effects. Second, scholars could investigate the effects of more discrete emotions. For example, frustration and anxiety might have different influences, although both are negative emotions. Third, according to personality systems interaction theory (Kuhl et al., 2021), the simultaneous fluctuation of both emotions has meaningful influences on the self-regulation process. Scholars could examine whether the changes in both emotions, as elicited by challenge stressors, influence job performance through this process. Fourth, scholars could try to compare the effects of emotions with those of motivations and cognitions, to see which mechanisms provide the best explanations.

The current study has some limitations. First, the cross-sectional research design makes it difficult to determine the causal relationships. Additional experimental or longitudinal designs would help test the underlying causality of the relationships examined (LePine et al., 2016; Van Laethem et al., 2019). A second limitation lies in the use of employees from three companies in China as the samples, as this may limit the generalizability of our results. The unique context of China may result in specific outcomes that would be different in other areas. People living in a strong collective culture (e.g., China) are more likely to conceal or mask their negative emotions because showing too many negative emotions (e.g., anger) commonly are considered to be a threat to the harmony of an organization (Kitayama et al., 2006), which may underestimate their effects. Third, this study only tested two specific types of challenge stressors, i.e., task complexity and time pressure; therefore, future research should pay more attention to other stressors such as work overload and a higher level of responsibility. Future research could also employ mixed methods, including both qualitative methods and quantitative designs, to properly develop and specify stressors based on different contexts (Liu et al., 2013).

In addition, we used the self-reporting method to measure the variables, which cannot precisely capture the construct. Although we used the marker variable technique (Lindell and Whitney, 2001) to alleviate the common source bias, and our results show there is not a serious threat, the self-reporting method may reduce the validity of the measurement. Accordingly, multiple sources of data should be considered in future research. For example, direct supervisors or/and coworkers should be invited to describe an employee’s job performance to avoid the common source bias. Finally, the measurement of the emotional states of employees could be influenced by events during the day in which the questionnaire was completed; therefore, an experience sampling method is recommended for future studies (LePine et al., 2005, 2016). For example, researchers could ask participants to report their daily work experience for 10 consecutive days, which could reduce the potential influence of specific work events.

## Conclusion

In conclusion, by combining the broaden-and-build theory of positive emotions (Fredrickson, 2001) and the transactional theory of stress (Lazarus and Folkman, 1984), our findings reveal that challenge demands (i.e., task complexity and time pressure) have a positive direct effect and offsetting indirect effects through positive and negative emotions on performance (i.e., task and contextual performance). The current study provides a novel perspective (i.e., the offsetting effect of positive emotions on negative emotions, including their relative specific and total indirect effects on the main effect) to explain why and how challenge stressors influence employees’ performance. Our findings also provide a practical tool for managers and organizations to manage and balance the work stress of employees by stimulating positive emotions to regulate negative emotions.

## Data Availability Statement

The data will be made available upon reasonable request.

## Ethics Statement

This study was reviewed and approved by the Research Committee at the Business School, Beijing Normal University. The patients/participants provided their written informed consent to participate in this study. All procedures performed in studies involving human participants were in accordance with the Ethical Standards of the Institutional and/or National Research Committee and with the 1964 Declaration of Helsinki and its later amendments or comparable ethical standards with written informed consent from all subjects.

## Author Contributions

QW, AX, WZ, and JQ contributed to conception and design of the study. JQ organized the database. QW and AX performed the statistical analysis and contributed most the first draft of the manuscript. WZ, XZ, and XT wrote sections of the manuscript. QW and WZ was responsible for the revision of the manuscript and led the R&R process. ZC and JZ gave critical help for the revision. AX, XZ, XT, and JQ also contributed to manuscript revision, read, and approved the submitted version. All authors contributed to the article and approved the submitted version.

## Conflict of Interest

The authors declare that the research was conducted in the absence of any commercial or financial relationships that could be construed as a potential conflict of interest.

## Publisher’s Note

All claims expressed in this article are solely those of the authors and do not necessarily represent those of their affiliated organizations, or those of the publisher, the editors and the reviewers. Any product that may be evaluated in this article, or claim that may be made by its manufacturer, is not guaranteed or endorsed by the publisher.
